# New Insights into Drug Reaction with Eosinophilia and Systemic Symptoms Pathophysiology

**DOI:** 10.3389/fmed.2017.00179

**Published:** 2017-12-04

**Authors:** Philippe Musette, Baptiste Janela

**Affiliations:** ^1^Dermatology Department, Rouen University Hospital, Rouen, France; ^2^Singapore Immunology Network, Singapore, Singapore

**Keywords:** eosinophil, drug-induced eruption, virus replication, cytokines, side effects

## Abstract

Drug reaction with eosinophilia and systemic symptoms (DRESS), also known as drug-induced hypersensitivity syndrome, is a severe type of cutaneous drug-induced eruption. DRESS may be a difficult disease to diagnose since the symptoms mimic those of cutaneous and systemic infectious pathologies and can appear up to 3 months after the initial culprit drug exposure. The symptoms of DRESS syndrome include rash development after a minimum of 3 weeks after the onset of a new medication, associated with facial edema, lymphadenopathy, and fever. Biological findings include liver abnormalities, leukocytosis, eosinophilia, atypical lymphocytosis, and reactivation of certain human herpes viruses. In DRESS, liver, kidneys, and lungs are frequently involved in disease evolution. Patients with serious systemic involvement are treated with oral corticosteroids, and full recovery is achieved in the majority of cases. DRESS is a rare disease, and little is known about factors that predict its occurrence. The key features of this reaction are eosinophil involvement, the role of the culprit drug, and virus reactivation that trigger an inappropriate systemic immune response in DRESS patients. Interestingly, it was evidenced that at-risk individuals within a genetically restricted population shared a particular HLA loci. In this respect, a limited number of well-known drugs were able to induce DRESS. This review describes the up-to-date advances in our understanding of the pathogenesis of DRESS.

## Introduction

Drug reaction with eosinophilia and systemic symptoms (DRESS) is a severe cutaneous drug-induced eruption (DIE) characterized by a virus like clinical presentation. Typically, the patient presents fever, lymphadenopathy, facial edema, and a maculopapular rash. Systemic involvement includes hepatitis and interstitial pneumonia. Severe renal and cardiac [eosinophilic myocarditis (EM)] involvement may be also found. Since DRESS is triggered by long-term drug exposure, it is essential to seek and identify the culprit drugs in the months prior to eruption. Other severe DIEs include Stevens–Johnson syndrome (SJS) and toxic epidermal necrolysis (TEN), both characterized by skin detachment. They occur after a short drug exposure and do not present systemic involvement (1). Non-severe DIE characterized by a benign maculopapular eruption does not present systemic signs or skin detachment. On the one hand, the sequence of immunological and biological events at the onset of DIE that play a key role in the pathogenesis may be shared between the three different forms of severe DIE; on the other hand, specific clinical manifestations may be influenced by patient-intrinsic genetic factors or external factors such as viral infection or reactivation that are not yet clearly identified and deciphered. DRESS may be difficult to diagnose and identify because symptoms evidenced could mimic several other diseases including infectious diseases and can appear a long time after initial culprit drug exposure. The RegiSCAR criteria were created to better evidence DRESS in drug-treated patients presenting a DIE (2). RegiSCAR is based on seven independent parameters and three of them are required (fever > 38°C, acute skin rash, lymphadenopathy, internal organ involvement, blood count abnormalities including atypical lymphocytes and eosinophilia) for the diagnosis of DRESS. Other criteria were developed in Japan: the Japanese consensus group diagnostic criteria for drug-induced hypersensitivity syndrome (3). These new diagnostic criteria require that a minimum of seven of nine symptoms be found to diagnose DRESS [skin eruption a minimum of 3 weeks after starting medication, symptoms not stopped when the drug is discontinued, fever, liver biological abnormalities, circulating leukocyte abnormalities including leukocytosis, atypical lymphocytosis, eosinophilia, lymphadenopathy, and reactivation of human herpesvirus 6 (HHV-6)] (Table S1 in Supplementary Material). In DRESS, the organs frequently involved are liver, kidneys, and lungs, and usual blood abnormalities include eosinophilia, atypical lymphocytes, and lymphocytopenia. Interestingly, a limited number of well-known drugs mainly including anticonvulsants are able to induce DRESS (Table 1). Patients with DRESS are usually treated with immunosuppressive drugs including mainly systemic corticosteroids, whereas usage of intravenous immunoglobulin is controversial. Full recovery is achieved in 90% of patients (4).

**Table 1:** DRESS inducers.

**Anticonvulsant**

Carbamazepine
Lamotrigine
Oxcarbamazepine
Phenobarbital
Phenytoin

**Antibiotic**

Minocycline
Sulfalazine
Sulfamethoxazole
Vancomycin

**Others**

Abacavir
Allopurinol
Dapsone
Mexilletine
Nevirapine
Salazosulfapyridine
Strontium ranelate

*Adapted from Cacoub et al. (4)*.

Drug-specific T-cells have been identified in patients and are supposed to be the primary effectors of the pathology in DIE patients (5). However, T-cells derived from healthy donors can also be activated with drugs without previous drug exposure (6–8). These interesting data could predict a higher occurrence of DIE in patients taking drugs than observed in real life. All the factors that may identify “at-risk” individuals in patients exposed to drugs are not yet determined. Interestingly, some genetic risk factors of DIE are associated with different HLA loci (9–14), these findings of primary importance cannot account alone for DIE occurrence, because HLA risk alleles are considered neither fully necessary nor fully sufficient for disease development (10). Interestingly, a relationship was also clearly evidenced between DIE and virus infection or reactivation. In this respect, endogenous herpes virus (HSV) can be reactivated and presented to the immune system in DRESS patients (15). However, there is no evidence that reactivation of HSVs can also occur in other DIE such as SJS and TEN, despite some isolated clinical cases, whereas some virus and mycoplasma induced eruptions may mimic SJS and TEN (16). Recurrence of DRESS with unrelated drugs can be observed in 25% of cases, whereas very little or no recurrence is found with TEN and SJS patients (17). There may also be factors related to the nature of the culprit drug, severe systemic involvement has been associated with allopurinol and minocycline, and prolonged evolution with non-Caucasian ethnicity and minocycline (18, 19).

## Pathophysiology

### The Hapten Theory and p-i Concept

A hapten is a small non-immunogenic molecule that becomes antigenic when it is bound to a carrier protein [reviewed in Ref. (20)]. By contrast, pro-hapten molecules require metabolization to become immunogenic and to be able to bind to proteins. Since detoxification enzymes are expressed by all patients, it has been proposed that detoxification enzyme polymorphisms could be responsible for the development of DIE and DRESS in only a subgroup of patients. However, no such polymorphism has been identified yet in patients with DIE (20). Indeed, the majority of small drug molecules can be recognized by the human immune system including T-cells despite lacking hapten structure (21). The direct binding of drugs and their metabolites to HLA that trigger T-cell responses has been called “p-i” concept (pharmacological interaction of drugs with immune receptor) (22). T-cells isolated from healthy donors and patients present the capacity to be stimulated by certain drugs indicating that some individual susceptibility factors are required to mount a pathological immune response (20).

### Drug Interactions with HLA Type

Very interesting results have been obtained showing that specific HLA variants are responsible for very high increased risk of DRESS or hypersensitivity occurrence. The first study to show a clear relationship between DIE and HLA subtype was performed in 2002 (11), the authors identified a very strong link between HLA-B*5701 in HIV-positive caucasians and the development of hypersensitivity to abacavir (*p* < 0.0001). The specific mechanism of T-cell activation by abacavir in the HLA groove was then identified in 2012 (23). The results were largely confirmed by other teams (24). Abacavir was found able to bind noncovalently and specifically to the peptide-binding groove of HLA-B*5701 molecule. The presence of the abacavir molecule in the groove induces a change in the repertoire of peptide presentation and as a consequence a T-cell response against a HLA/self-peptide complex. Those modifications of the immune presentation of endogenous and self proteins induce an important inflammatory response that triggers systemic clinical and biological signs. Interestingly, they also evidenced that the non-covalent abacavir binding to the HLA groove modified the self-peptide repertoire presented in the groove and represented a possible immunological mechanism of autoimmunity that can appear after DIE (24, 25). In this respect, some cases of autoimmune diseases including diabetes and thyroiditis were evidenced in patients after occurrence of a DRESS (26, 27).

HLA links were also found for specific DRESS inducers. Carbamazepine is an anticonvulsant drug considered as a major DIE inducer. Susceptibility to carbamazepine reactions has been evidenced in patients with HLA-B*1502 variant (14). The mechanism of T-cell activation induced by carbamazepine is supposed to be the same as that described for abacavir (23). HLA-B*1502 is commonly found and exclusive to South East Asia populations. In contrast, carbamazepine immune response in European populations is associated with the presence of HLA-B*3101 (9). Allopurinol induces reactions in HLA-B*5801 patients (12). All these HLA associations probably share a similar T-cell immune activation mechanism that depends on culprit drug/HLA interaction and HLA/peptide repertoire presentation. The identification of DIE risk-associated HLA variants opens new avenues for physicians by using patient stratification for DIE risk using HLA typing. In a prospective study, carbamazepine was not used in Taiwanese patients carrying the HLA-B*1502 variant (28). By determining HLA phenotype before drug introduction, the incidence of DIE was dramatically reduced, since none of the 4,120 HLA-B*1502 negative included patients developed SJS, TEN, or DRESS compared to an estimation of 10 expected SJS and TEN cases. Mild rash was found in 6% of the non-HLA-B*1502 patients. These results highlight the major role of the major histocompatibility complex in DIE, whereas additional risk factors for benign eruption may play a role in 6% of the population. In conclusion, for certain drugs and particular populations, screening patients HLA haplotype before drug introduction could be used to reduce DIE occurrence (29).

### Antiviral Responses

**Figure 1:**
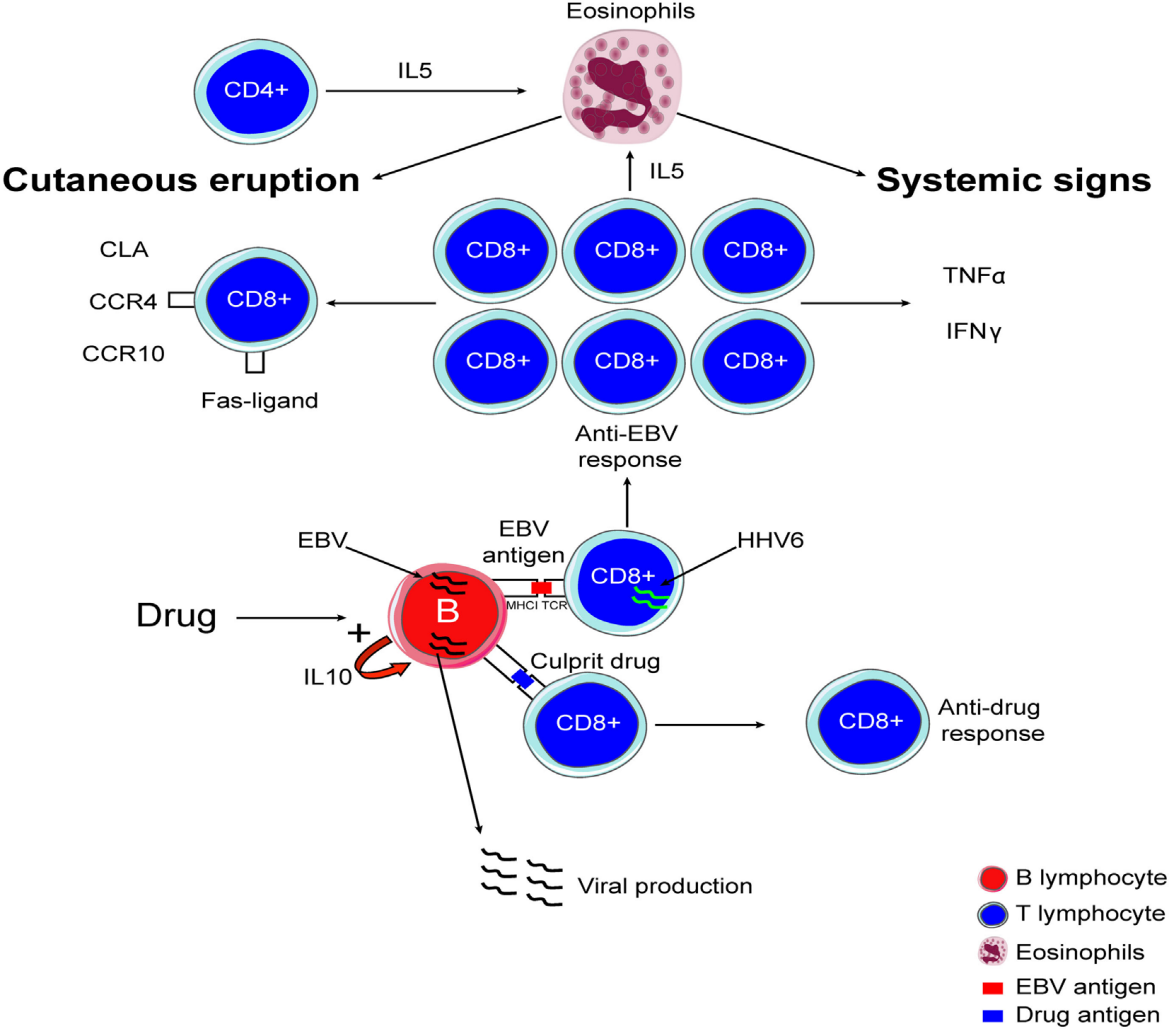
Immunological mechanism involved in drug reaction with eosinophilia and systemic symptoms.

It is now largely accepted that DRESS can be associated with reactivation of inactive viruses, especially in individuals infected with members of the human herpes viridae family including mainly HHV-6, EBV, and CMV (30–35). As a consequence, we proposed that viruses may play a key role in DRESS pathogenesis. Interestingly, HHV-6 and EBV both induce a disease associated with fever and skin rash. HHV-6 is able to infect T-cells (36) and to dysregulate CD8^+^ lymphocytes by inducing abnormal expression of CD4 that may increase T-cell activation and antiviral response (37). Picard et al. evidenced a massive anti-HSV T-cell response in the blood and involved organs of DRESS patients (15). In this study, 40 cases of DRESS were analyzed; Picard and colleagues showed that circulating EBV-specific CD8^+^ T-cells were expanded within the T-cell population, representating up to 21% of the total cytotoxic T-cell population in DRESS patients compared with <0.1% in control patients. Activated T lymphocytes produced large amounts of TNFα, IL-2, and IFNγ, considered as key mediators of the cytokine release that induces the symptoms found in DRESS patients. Interestingly, EBV-specific T lymphocytes were detected in affected organs in DRESS patients including liver, skin and lungs. Moreover the authors demonstrated that the culprit drug is able to induce *in vitro* viral reactivation (15). In this respect, reactivation of the HSV that triggers uncontrolled antiviral T lymphocyte response leads to systemic inflammation associated with organ failure. These immunological events may represent a specific feature of DRESS compared to other DIE (Figure 1). In addition, IL-10 secretion by B cells and inflammation may promote viral reactivation. The inflammation induced by the virus and the systemic inflammation found in DRESS may represent a loop that induces a long lasting inflammation process. In this respect, multiple HHV family member reactivations were identified in DRESS patients (15, 35, 38, 39). By contrast, HSV reactivation or infection in SJS and TEN patients is not proven (40, 41). Further investigation is now required to decipher the mechanisms and roles of the culprit drug-specific T lymphocytes response in DRESS patients, in order to better understand the role of culprit drugs on the onset and the amplification of anti-HSV immune responses. Interestingly, expansion of regulatory T-cell populations (T-reg) can be found in DRESS patients (42). This phenomenon could also play a role in infection or reactivation of HHV- 6 (43). Altered function of T-reg may also plays a role in the occurrence of autoimmune disease evidenced in DRESS patients after initial DIE.

### Eosinophilia

Cutaneous DIEs are usually associated with eosinophilia, and cutaneous eosinophil infiltration plays a key role in cutaneous eruption. Interestingly, cutaneous eosinophil infiltration is more pronounced in DRESS (44). For DRESS, eosinophilia is a diagnosis criterion and is found in 80% of patients (4). The number of eosinophils is increased in blood and in skin and involved organs, whereas in physiologic conditions eosinophils are not present in skin, liver, and lungs. Eosinophils are circulating granulocytes involved in the host defense against parasites, bacteria, viruses and in allergic reactions. Eosinophils are also involved in diverse inflammatory responses and can regulate innate and adaptive immunity. Eosinophils are derived from bone marrow precursor and differentiate mainly in response to IL-5. After, they enter the peripheral blood and circulate. Finally, eosinophils enter and home into tissues following an eotaxin gradient. Within the tissue, they can develop an extracellular trap formation. IL-5 again is a key cytokine for eosinophil survival, proliferation, and activation (45). Both CD4^+^ and CD8^+^ T-cells are thought to be involved in IL-5 production prior to eosinophil recruitment. The main factors in DRESS that activate and recruit eosinophil are IL-5 and eotaxin. In synergy with IL-5, eotaxin-1 has been identified to be a very selective and potent recruiter for eosinophil (46, 47). Eotaxin-1 is a CC chemokine, also known as cysteine cysteine ligand 11 (CCL11). Under basal conditions or during allergy and inflammation, eotaxin *via* interaction with its receptor CCR3 acts in synergy with IL-5 to recruit eosinophils into tissues. Interestingly, an increase in serum eotaxin level has been highlighted during the course of DRESS syndrome and eotaxin, in synergy with IL-5 has been identified as a key player in activating and recruiting eosinophils in drug-induced cutaneous eruption (47).

**Figure 2:**
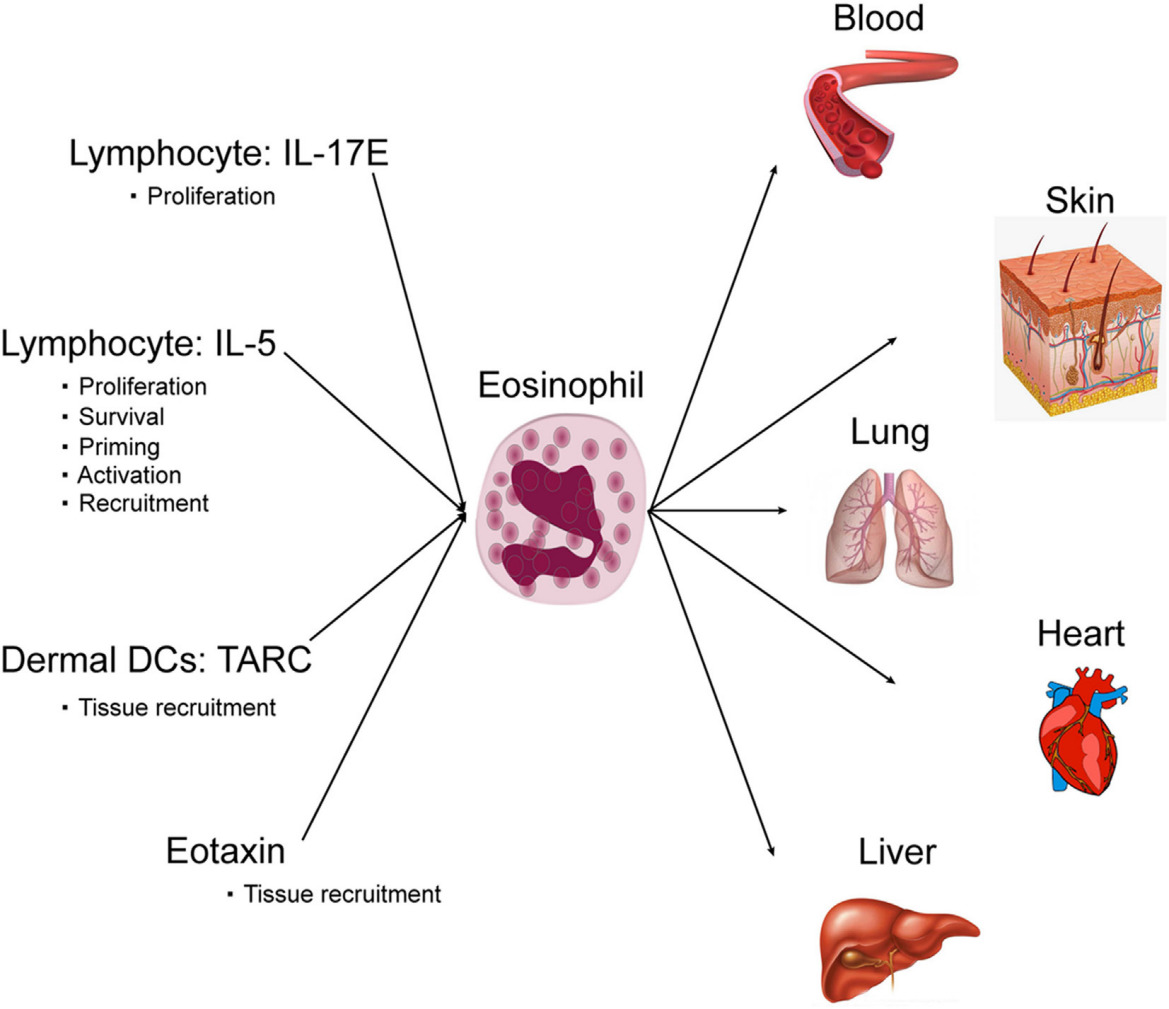
Role of eosinophils in drug reaction with eosinophilia and systemic symptoms.

In addition to IL-5 and eotaxin, eosinophil migration from circulation can also be controlled by thymus activation-regulated chemokine (TARC/CCL17) (48). TARC is a member of the CC chemokine family that is constitutively expressed in the thymus. It is the ligand of CCR4 that is expressed mainly by Th2 lymphocytes, basophils, and natural killers and is also produced by endothelial cells, bronchial epithelial cells, fibroblasts, keratinocytes, and dendritic cells (48). The pathogenic role of TARC has been highlighted in skin diseases such as atopic dermatitis, and bullous pemphigoid. TARC is known to be present in cutaneous lesions massively infiltrated by eosinophils; moreover, serum TARC levels reflect disease activity (48). In addition to the activity of attraction of Th2 lymphocytes, studies have shown that TARC is a potent eosinophil chemoattractant and has been associated with eosinophilic pustular folliculitis, highlighting a correlation between serum TARC levels and peripheral blood eosinophil number (48). Interestingly in drug eruption, a strong correlation between serum TARC levels and blood eosinophil count has been highlighted. Serum TARC levels during the acute phase were higher in DRESS patients compared with SJS/TEN patients or in cases of benign maculopapular eruption. TARC levels were correlated with the occurrence of skin eruptions, serum IL-5 levels and eosinophil counts (49). It has been demonstrated that the CD11c^+^ dermal dendritic cells in DRESS patients may be the main source of TARC. Interestingly, due to 100% sensitivity and 92.3% specificity in diagnosing DRESS and elevated levels observed in the serum especially at the early stage of DRESS, serum TARC measurement could even be a potent diagnostic value for DRESS among patients with various types of drug eruptions (49) (Figure 2).

Interestingly, we found in DRESS patients an over expression of IL-17 including IL-17E (IL-25) that play a key role in eosinophil blood increase (15). IL-17E over expression may increase circulating eosinophils, IL-4, IL-5, eotaxin, and IgE. As a consequence, IL-17E may play a key role in the control and amplification of the eosinophilic immune responses found in DRESS patients (50).

### Damage Induced by Eosinophilia

In multiple target tissues, eosinophils specifically eliminate antibody bound parasites through the release of cytotoxic granule proteins (45). Therefore, cytotoxic release produces organ damage. The most dangerous involvement in DRESS patients is caused by heart eosinophil damage.

Eosinophilic myocarditis is a rare and potentially fatal condition if left untreated. EM can have a delayed presentation and can appear even after a long delay. Delayed corticosteroid treatment can result in heart failure and death. Cardiac involvement must be detected early by echocardiography, and elevated serum troponin since ECG signs may not be present and may be evidenced too late. Eosinophil toxicity may also involve lung causing interstitial pneumonitis detected by early chest radiography. Interstitial pneumonitis requires systemic steroid treatment. Hepatitis, detected in blood by hepatic enzyme increase, is a diagnostic criterion of DRESS. In rare cases, hepatic involvement may lead to fulminant hepatitis with dramatic consequences including severe hepatic failure. In a limited number of cases of severe hepatic failure, hepatic transplantation may be required. In severe hepatic failure, steroid usage is debated. In diverse organs including digestive tract, thyroid, and central nervous system, nerves may also be infrequently involved (Figure 2).

Finally, eosinophil activation and multiplication is related to antiviral and culprit drug immunological response leading to organ eosinophil infiltration. Granule release represents a key factor of tissue damage in DRESS patients.

In conclusion, DRESS is a systemic drug reaction wherein eosinophil activation and multiplication is driven by an immunological response directed against viral reactivation and a culprit drug. Eosinophils infiltrate organs in response to chemokines including eotaxin-1 and TARC, in synergy with IL-5, and granule release represents a key factor of tissue damage.

## Author Contributions

BJ and PM wrote the manuscript and made the figures.

## Conflict of Interest Statement

The authors declare that the research was conducted in the absence of any commercial or financial relationships that could be construed as a potential conflict of interest.
